# Assessing neo-natal mortality trends in Pakistan: an insight using equity lens

**DOI:** 10.1186/s13690-021-00767-1

**Published:** 2022-01-04

**Authors:** Zainab Dawood, Naeem Majeed

**Affiliations:** Department of Public Health, University of the Punjab, Lahore, Pakistan

**Keywords:** Neonatal mortality, Equity, Pakistan, Wealth quintiles, Urban rural divide

## Abstract

**Background:**

Almost 2.5 million neonates died in the first year of life in the year 2017. These account for almost half of the total deaths of children under the age of 5 years. Overall, child mortality has declined over the past two decades. Comparatively, the pace of decline in neonatal mortality has remained much slow. Significant inequalities in health across several dimensions – including wealth, ethnicity, and geography – continue to exist both between and within countries, and these contribute to neonatal mortality. This study aims to quantify the magnitude of inequalities in neonatal mortality trends by wealth quintile and place of residence with province wise segregation.

**Methods:**

The study was done using raw data from the last three Pakistan Demographic & Health Surveys (2017–18, 2012–13 and 2006–07). The concentration curves were drawn in Microsoft Excel 365 using scatter plot as graph type while the frequencies were calculated using SPSS 24.

**Results:**

The situation of inequity across provinces and in rural vs urban areas has slightly declined, however, gross inequities continue to exist.

**Conclusions:**

Presentation of outcomes data, such as neonatal mortality in various wealth quintiles is an effective way to highlight the inequities amongst income groups as it highlights the vulnerable and at-risk groups. In other countries, rural-urban distribution, or ethnic groups may also reflect similar differences and help in identifying high-risk groups.

## Background

It was estimated that over 141 million newborns will be added to the world population in the year 2019, the number rising with every year. On the other hand, almost 2.5 million died in the first 28 days of life in the year 2017 [1]. These 2.5 million deaths account for 47% of the total deaths in children under the age of 5 years, increasing from 40% in 1990 [2]. Although the overall under five child mortality has declined over the past decade or so, the decline in the neonatal mortality has not been at the same pace, according to the United Nations’ Interagency Group for Child Mortality Estimation (UNIGME) [2]. This rise in the proportion of neonatal deaths was expected, as predicted in earlier studies which mentioned that the infant mortality rates are expected to decline due to widespread implementation of known and cost-effective interventions like vaccines for preventable diseases, and oral rehydration therapy for diarrhea. These interventions resulted in a decline in mortality beyond the neonatal period, thereby shifting the proportianate burden of mortality more towards neonatal deaths [3].

Almost 75% of the newborn deaths happen during the first 7 days of life, while over 25% happen before completion of the first 24-h of life, highlighting the fact that the probability of surviving increases with every passing day for the newborn [4].

There is increasing evidence that significant inequalities in health across a number of dimensions – including wealth, ethnicity, and geography – continue to exist both between and within countries [5, 6]. Indeed, it has been suggested that inequalities may have widened in recent times [7, 8]. World Health Organization (WHO) defines health inequities as “Health inequities are differences in health status or in the distribution of health resources between different population groups, arising from the social conditions in which people are born, grow, live, work and age. Health inequities are unfair and could be reduced by the right mix of government policies” [9].

Over 65% of the total neonatal deaths occurring globally belong to just 10 countries. Most of these countries are from Asia. Pakistan ranks third amongst these ten. It is estimated that almost 300,000 newborns die annually in the country. The latest reported neonatal mortality rate is 42 per thousand live births, accounting for around 7% of neonatal deaths happening globally [10–14]. Pakistan is a signatory to the Alma Ata Agreement [15], which stipulates that health is affected by social position and the underlying inequality in a society, and this needs to be addressed.

Equity in health is a basic and core principle of the “Primary Health Care approach” [16]. It is, henceforth, implicitly or explicitly mentioned in the health policies of a majority of the countries [17]. It is widely recognized that people’s equitable access to health care services is vital to sustaining good health which depends primarily on income levels and the cost and availability of quality health services. There is an established correlation between social and health inequality. While inequalities are ubiquitous and a reality of life, the concept of inequity refers to the degree of unfairness and injustice in societies which often result from pervasive inequalities [18].

The Sustainable Development Goals (SDGs) make up a set of targets developed in a holistic manner to guide future development projects. Health is at the center of the third SDG, to “Ensure healthy lives and promote well-being for all at all ages” [19]. This goal also focuses on the integration of equity, human rights, gender, and social determinants into policies. These programs and institutional mechanisms are needed all over the world, as they are are vital for empowering women and men, and for reducing inequities between and among populations, thereby promoting everyone’s human rights. Goal 10 (reduce inequalities) also calls for a reduction in inequality within and among countries to empower and promote the inclusion of all [20]. New-born health has also been taken seriously as the target for Neonatal Mortality Rate (NMR) for each country has been set at 12 per 1000 live births under the SDG 3 i.e. Good Health and Well-Being [19].

Human welfare and development is a basic right of each individual as highlighted in the Constitution of Pakistan [21]. Access to majority of the curative and preventive health services, and hence, their utilization is not equitable in Pakistan. These inequities are specifically intensified as we move from the richest towards the poorest wealth quintiles, as well as across various geographical regions of the country, especially highlighting the rural and urban divides. A multitude of initiatives have been taken over time to improve maternal and neonatal health indicators across the country. However, the progress so far is sluggish, and the inequities amongst different wealth and income statuses, social statuses, literacy rates and geographies have remained more or less constant [15, 22].

Using national data from Pakistan, this study aimed to:

- Observe trends in neonatal mortality in Pakistan with an equity lens; utilizing data from the last three Demographic and Health Surveys
- Quantify the magnitude of inequalities in neonatal mortality trends by wealth quintile and place of residence with province wise segregation.

## Methodology

The study was conducted using raw data from the last three Pakistan Demographic & Health Surveys (PDHS) i.e., 2017–18 [10], 2012–13 [23] and 2006–07 [24]. The 2017–18 PDHS is the fourth in the series of Demographic and Health Surveys (DHS) conducted so far in Pakistan. The earlier three PDHSs were carried out in the years 1990–91, 2006–07 and 2012–13. These surveys were carried out with collaboration of National Institute of Population Studies (NIPS), Islamabad, Pakistan and the DHS Program (of the United States Agency for International Development, USAID). Raw data files used for the purpose of this study was “Birth Recode”, downloaded from the DHS website.

### Variables used for analysis

The variables used for neo-natal mortality was “Age at death (months-imputed)”. This variable was recoded into new variable (deaths at 0 months taken as “yes” for neo-natal mortality and all others taken as “no” for neo-natal mortality). Data was filtered by the variable “births in last 5 years” and the cases weighted by “women’s individual weight” using the same methodology as used for measurement of neonatal mortality in the PDHS report. Other variables used for the purpose of analysis were “province”, “area of residence” and “wealth quintile”.

### Measurement of inequities through concentration curves

Inequities were observed in neonatal mortalities in different wealth quintiles by geography and type of residence i.e., national and provincial, rural and urban.

Several methods have been in use to date for measurement of inequities. Some have their origin in research on income inequality (e.g. Lorenz curve and the associated Gini coefficient) or from modifications of these (e.g. concentration index). Other methods are based on measures of association (index of dissimilarity, slope index of inequality). We used the concentration index and corresponding concentration curve.

### Concentration curves and index

The concentration curves and index have their origin in research on income inequality (e.g. Lorenz curve and the associated Gini coefficient) or from modifications of these (e.g. concentration index).

Inequities are represented by concentration curves that are relatively easier to understand compared to the concentration indices. The concentration curve plots the cumulative proportion of the individuals under consideration ranked by wealth against the cumulative proportion of the health/healthcare variable (e.g. antenatal care visits, skilled birth attendant etc.) being measured. If the health indicator under consideration is an undesirable outcome, a concentration curve that lies above the line of equality signifies inequity disfavoring the poor and is bad from the equity point of view. If the indicator being considered is a desirable one (e.g. immunization coverage), a concentration curve that lies above the diagonal (line of equality) shows inequity favoring the poor – a situation that is desirable from the equity point of view. The concentration index (C) is computed in a spreadsheet program using the following formula:

$$ C=\left({p}_1\kern0.5em {L}_2-{p}_2\kern0.5em {L}_1\right)+\left({p}_2\kern0.5em {L}_3-{p}_3\kern0.5em {L}_2\right)+\dots +\left({p}_{T-1}\kern0.5em {L}_{T-p\kern0.5em T}\kern0.5em {L}_{T-1}\right) $$

Where *p* is the cumulative percent of the sample ranked by economic status, *L(p)* is the corresponding concentration curve ordinate and *T* is the number of socioeconomic groups [25].

The concentration curves were drawn in Microsoft Excel 365 using scatter plot as graph type while the frequencies were calculated using SPSS 24.

### Sampling

The sample size was 22,610 births (2006–07), 27,320 (2012–13) and 22,378 (2017–18) out of which 1328, 1703 and 1079 neonatal deaths were observed respectively.

All cases having births in the last 5 years preceding the survey were included in the sample, and these cases were filtered from the raw-data using the variable “births in last five years preceding the survey”. Cases belonging to areas other than the four provinces were excluded from the study as Islamabad Capital Territory (ICT) and Gilgit Baltistan (GB) data was not available separately for PDHS 2006–07 while data for GB and Federally Administered Tribal Area (FATA) was not available separately for PDHS 2017–18. Among these four provinces, Punjab has the highest population, while Balochistan is the largest province by area. Punjab ranks highest in terms of development, literacy rates and health indicators, followed by Sindh, Khyber Pakhtunkhwa (KP), and Balochistan according to surveys like the Pakistan Demographic & Health Survey used in this study.

## Results

The data analysis revealed that out of the total births observed in the sample for 2006–07 PDHS, 54% were from Punjab, 25% from Sindh, 16% from Khyber Pakhtunkhwa (KP) and 5% from Balochistan. Similar proportions were observed in the sample for PDHS 2012–13 and 2017–18 (Table 1). However, as far as the proportion of neo-natal deaths was concerned, 59% were from Punjab, 25% from Sindh, 13% from KP and 5% from Balochishtan in 2006–07 while 62% from Punjab, 22% from Sindh, 11% from KP and 5% from Balochistan. Thus, the share from Punjab and Balochistan increased while that from Sindh and KP decreased (Table 1). In general, a downward trend of neo-natal mortality is observed (reduced from 63 to 42 per 1000 live births according to PDHS 2012–13 versus PDHS 2017–18).

**Table 1:** Number of births and neo-natal deaths observed in PDHS 2006–07, 2012–13 and 2017–18

Variable	Region	Number and proportion of cases reported in each survey		
		PDHS 2006–07	PDHS 2012–13	PDHS 2017–18
Births	**Pakistan**	**22,610 (100%)**	27,320 (100%)*	22,378 (100%)*
	Punjab	12,258 (54%)	15,233 (55%)	11,156 (50%)
	Sindh	5699 (25%)	6405 (23%)	5544 (21%)
	KP	3569 (16%)	4180 (15%)	4100 (18%)
	Balochistan	1084 (5%)	1502 (5%)	1578 (7%)
Neo-natal deaths	**Pakistan**	**1328 (100%)**	1703 (100%)*	1061 (100%)*
	Punjab	787 (59%)	1059 (62%)	607 (57%)
	Sindh	328 (25%)	376 (22%)	218 (21%)
	KP	167 (13%)	185 (11%)	183 (17%)
	Balochistan	46 (3%)	83 (5%)	53 (5%)
Antenatal care coverage	**Pakistan**	**61%**	**73%**	**86%**
	Punjab	60.9%	77.8%	92.3%
	Sindh	70.4%	78.2%	85.7%
	KP	51.3%	60.5%	80.1%
	Balochistan	40.7%	30.6%	55.5%
Skilled attendance at birth	**Pakistan**	**39%**	**52%**	**69%**
	Punjab	37.7%	52.5%	71.3%
	Sindh	44.4%	68.5%	74.8%
	KP	37.9%	48.3%	67.4%
	Balochistan	23%	17.8%	38.2%
Vaccination coverage (BCG, at birth)	**Pakistan**	**80%**	**85%**	**88%**
	Punjab	85.5%	91.6%	96.5%
	Sindh	76.7%	78.5%	82.3%
	KP	71.1%	79.7%	81%
	Balochistan	63%	48.9%	46.6%

* Total figure for Pakistan, also includes GB, ICT and FATA which have not been included in the study as matching data is not available uniformly for the three surveys

**Fig. 1:**
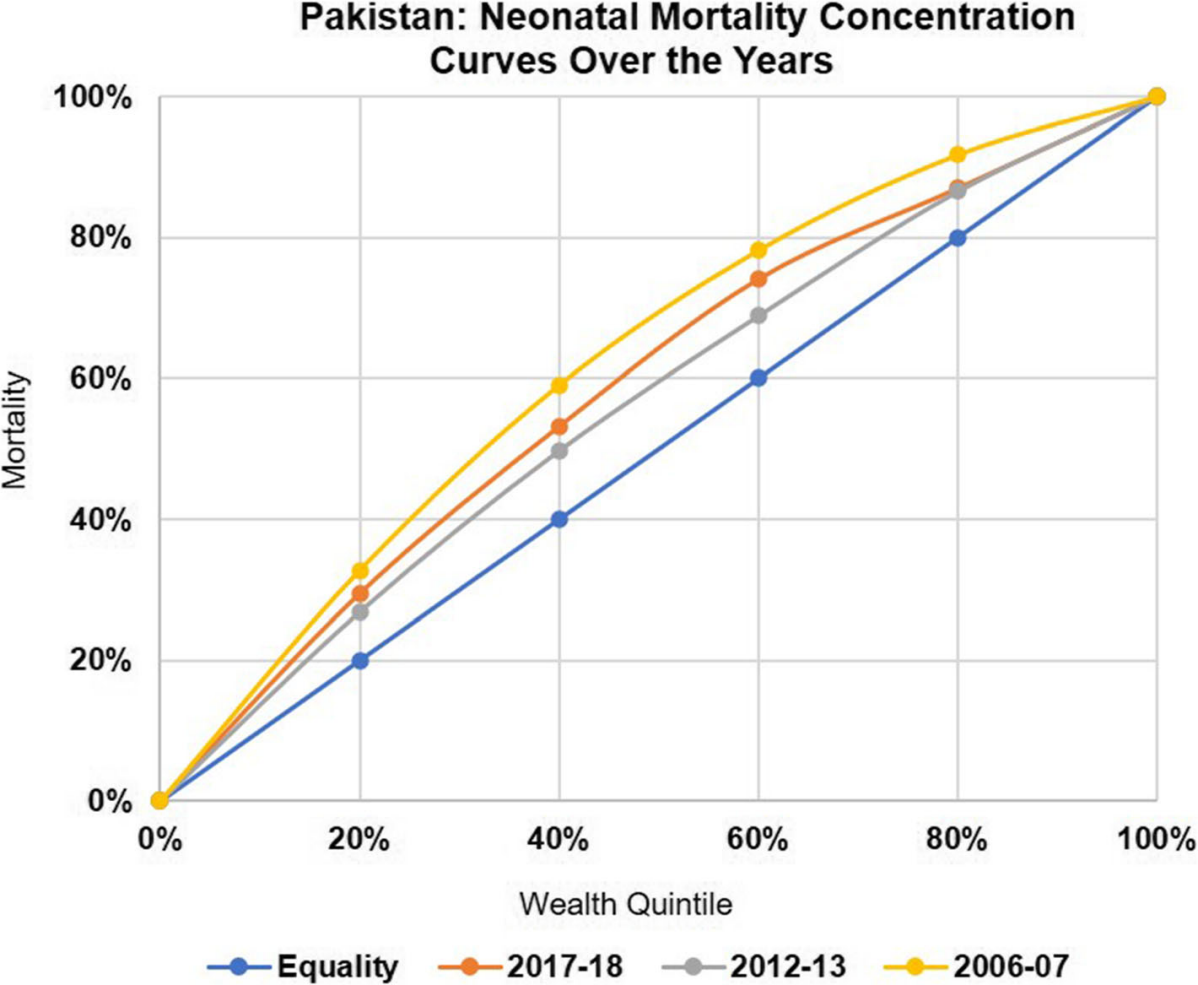
Pakistan: Neonatal Mortality Concentration Curves Over the Years

**Fig. 2:**
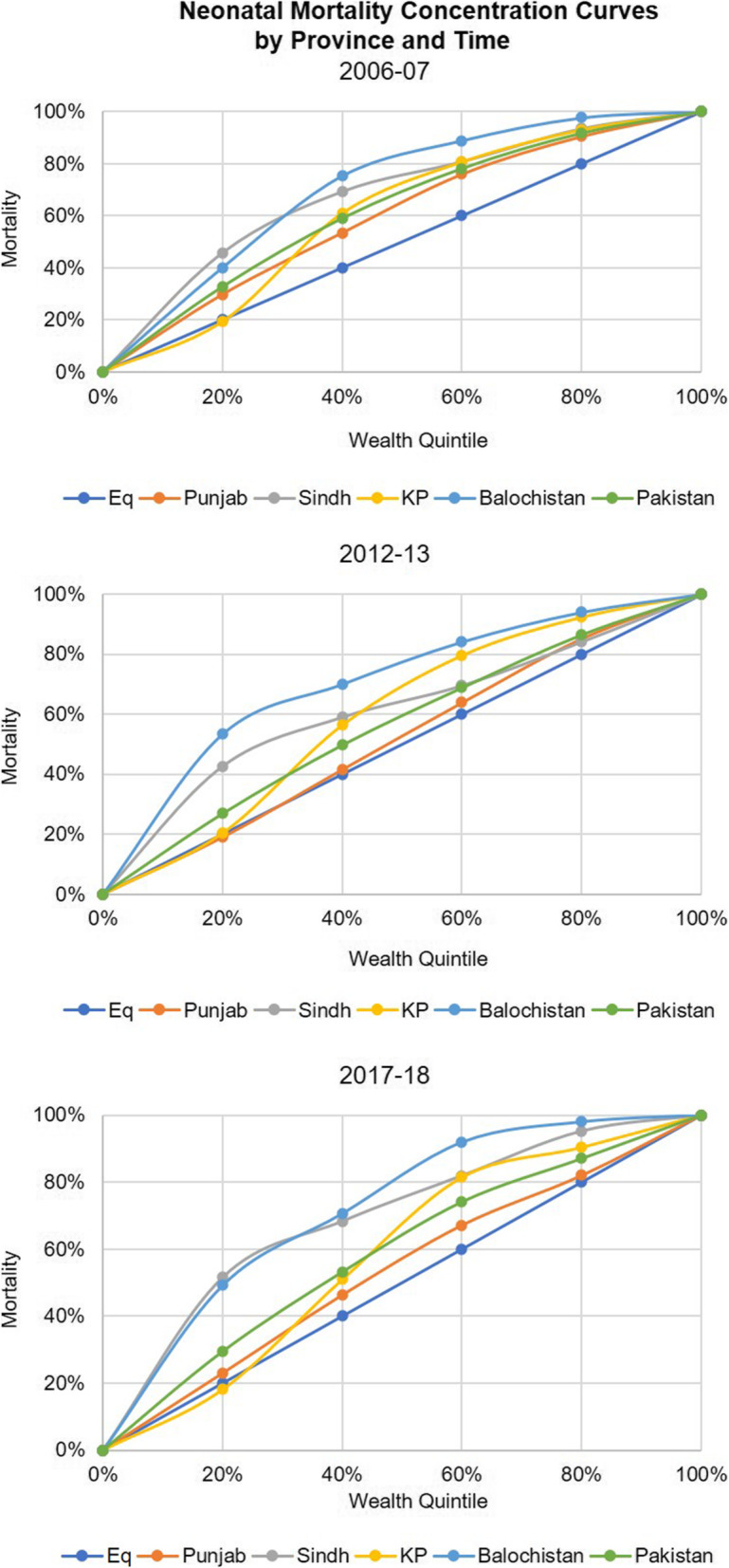
Neonatal Mortality Concentration Curves by Province and Time

When we compare the concentration index for neo-natal deaths by wealth quintile for Pakistan (Fig. 1), there was an overall decrease from − 0.247 to − 0.127^1^ from 2006 to 07 to 2012–13 reflecting an improvement in terms of equity as the gap between the number of deaths among the richest and poorest quintiles narrowed down. Same was observed in the provinces of Punjab, Sindh, and Khyber Pakhtunkhwa where the concentration index decreased from − 0.198 to − 0.038; − 0.357 to − 0.221 and − 0.216 to − 0.195 respectively. However, very minor decrease was observed in Balochistan i.e., from − 0.407 to − 0.406. Thus, the lowest level of inequity was observed in Punjab followed by KP, Sindh and Balochistan during the 2012–13 PDHS (Table 2, Fig. 2).

**Table 2:** Concentration index for neo-natal mortality by wealth quintiles

Area	Concentration Index for neo-natal mortality		
	2006–07	2012–13	2017–18
**Pakistan**	**−0.2472**	**−0.12759**	**−0.17531**
Punjab	−0.19847	−0.03841	−0.07374
Sindh	−0.35748	−0.22176	−0.38869
KP	−0.21621	−0.19529	−0.16307
Balochistan	−0.40744	− 0.4062	− 0.43876

**Fig. 3:**
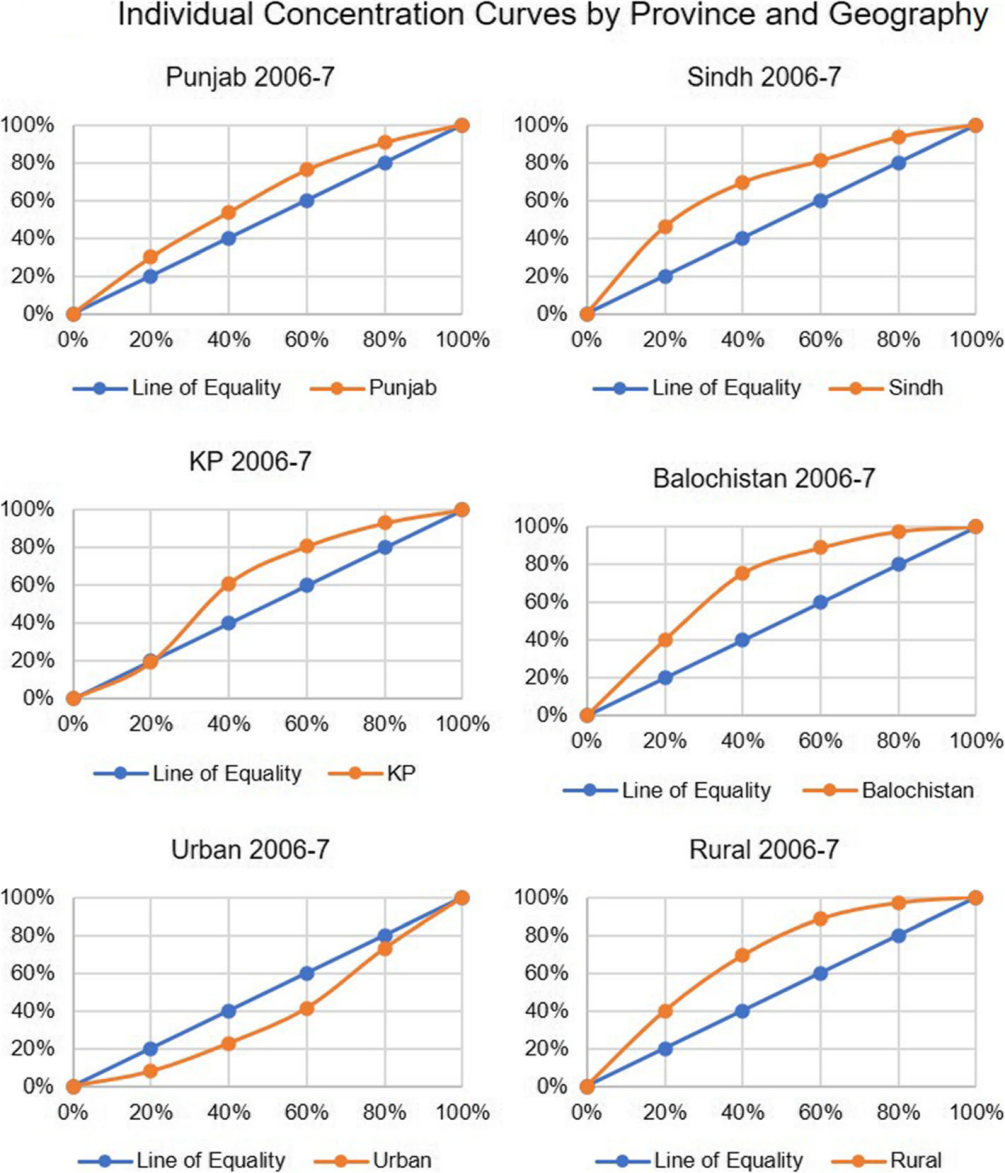
Individual Concentration Curves by Province and Geography

**Fig. 4:**
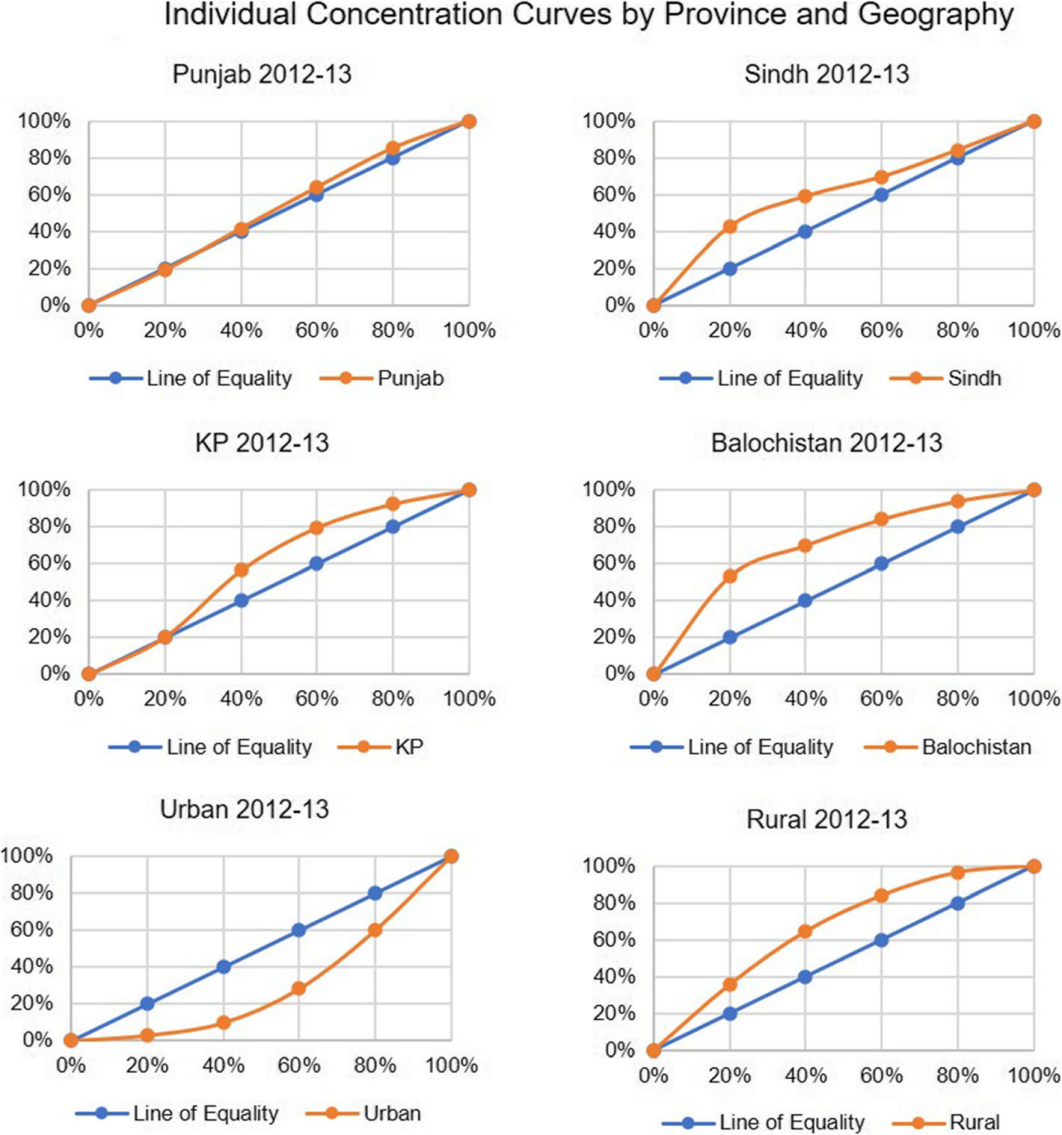
Individual Concentration Curves by Province and Geography

**Fig. 5:**
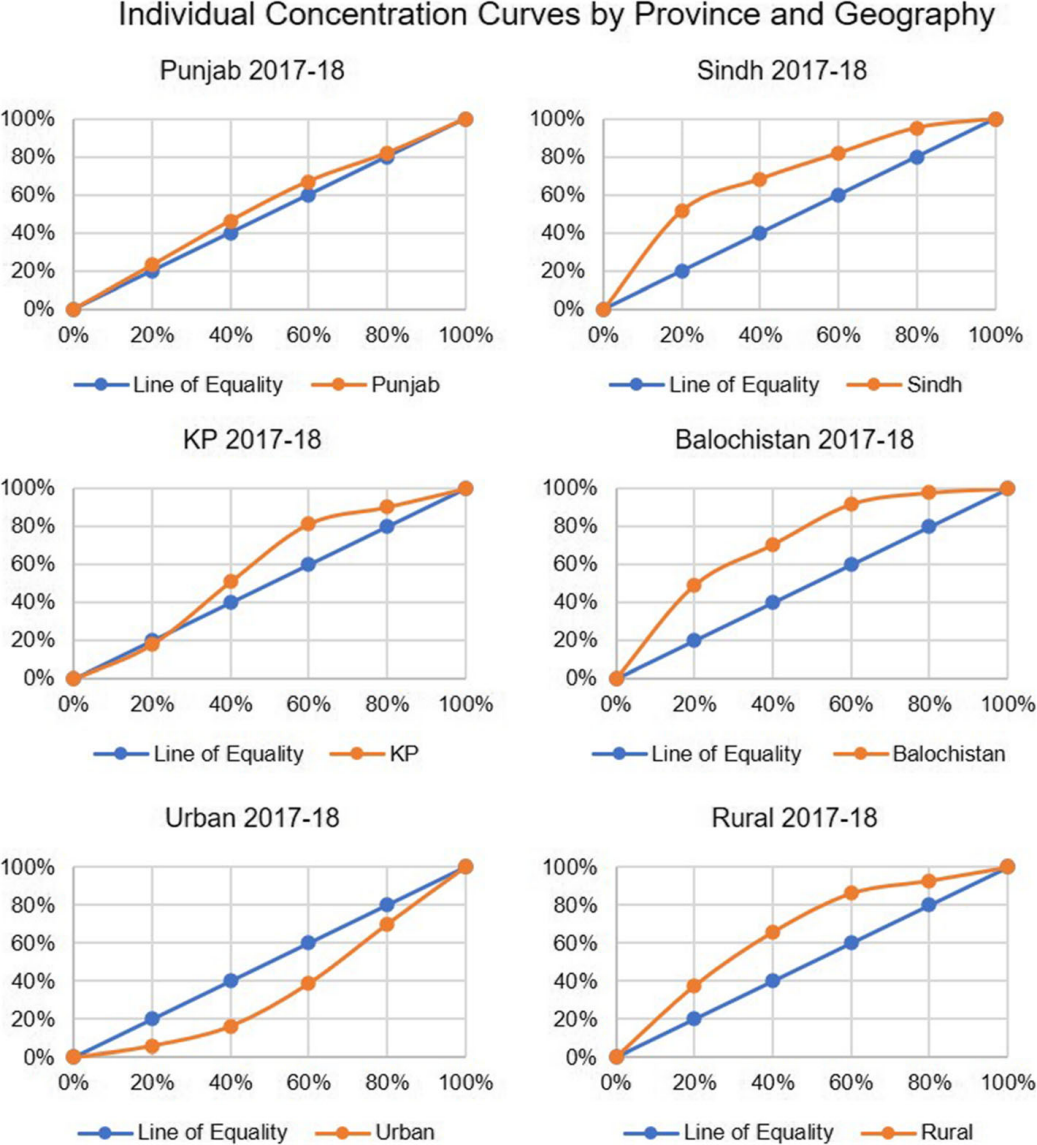
Individual Concentration Curves by Province and Geography

When we compare the concentration index for neo-natal deaths by wealth quintile for Pakistan, there was an overall increase from to − 0.127 to − 0.175 from 2012 to 13 to 2017–18 (Figs. 1 and 3) reflecting a decline in terms of equity as the gap between the number of deaths among the richest and poorest quintiles widened. However, if we compare 2017–18 with 2006–7 (Fig. 2), the gap has narrowed. In case of the provinces, Punjab and Sindh showed a rise compared to 2012–13 while Khyber Pakhtunkhwa showed a continuing declining trend and Balochistan a rising trend (Table 2, Figs. 4 and 5).

## Discussion

Disparities among the developed and under-developed or developing countries are gross, and the gap is gradually being widened in some areas; as is the case inter-country as well as intra-country. Children belonging to the poor quintiles are more vulnerable compared to their counterpart children belonging to richer quintiles, due to multiple challenges like malnutrition leading to weaker immunity, more exposure to risky and hazardous environments, and lesser or no access to both preventive and curative health. Efforts to improve the health outcomes through provision of subsidy for health services also usually doesn’t reach the vulnerable, and those not in need frequently benefit from such subsidies [26].

A study published in 2016 showed significant variation in coverage and inequalities across various regions of Afghanistan. These results are quite the expected ones, and highlight the fact that comparative availability and accessibility of health services is better in urban areas [18]. According to WHO, the disparity among high- and low-income countries for neonatal death is fairly large and it continues to increase. Another study states that, while there is little doubt that lower income is one of the major factors underlying inequitable access to services, the overall situation is quite complicated; as multiple factors like geography, economy and ethnicity exist as determinants of inequity, and their simultaneous presence in certain cases makes the situation complex. Further to this, the importance of one or the other determinant in relation to the other determinants may vary with the passage of time [27].

In an Ethiopian study, it was noted that geography over-rides the economic factors as the health facilities are at a distance from the population, making access difficult even for those who do not have economic issues. Thus, the risk of child (or newborn) mortality does not correlate with the income groups; and rather correlates with the type of residence i.e. rural versus urban [28]. Studies in India also showed that the underprivileged groups like the financially weak, poorly literate, or living in rural areas, had limited access to healthcare services, thus leading to poorer outcomes for health [29, 30]. Similar findings were observed in this study, in the case of Pakistan and for the provinces except for Balochistan where the inequities continue to prevail in the same ratio throughout the last decade. This is an important finding, for the Government as the number of health facilities in this province need to be increased to ensure adequate geographical coverage. The yardsticks for establishment of new health facilities in such areas should be geographical distances rather than population size.

A comparative study of 14 developing, low- and middle-income countries that had undertaken at least two Demographic and Health Surveys during the 1980s and 1990s was done by Minujin and Delamonica. These countries had shown progress in child survival and decline in mortality. Out of the 14 countries, broadening of gap in child mortality amongst the richest versus poorest wealth quintiles was observed in eight [31]. Most of these 14 countries have similar rural-urban and rich-poor divides as in Pakistan. PDHS for Pakistan shows that the gap between the richest and poor wealth quintiles has increased from 2012 to 13 to 2017–18; and the overall neonatal mortality has declined from 58 to 42 per 100,000 live births nationally during the same interval. This could be an alarming indicator for quality of services being provided even in the urban areas.

Geographic and ethnic disadvantages also result in lack of access to healthcare services; as much as the economic disadvantages. Therefore, health systems should consider these three determinants to provide preventive services like integrated management of newborn and childhood illnesses (IMNCI), vaccinations, and other interventions for newborn care through an equity-focused approach [6].

Inequities between the populations at the lower and higher end of the risk spectrum are further aggravated due to limited availability of effective interventions for the most marginalized children, as highlighted by Tugwell et al. [32]. Systematic reviews of demographic and health surveys show consistent inequities in child health across multiple countries [33, 34]. The study conducted in Afghanistan discussed that unfair inequities and inequalities in service provision, access to services, and product availability to different segments of the population lead to societal inequities [18].

Kruk et al. concluded that “redistributive health policies that promote pro-poor distribution of health services may reduce the gap in under five mortalities between rich and poor in low-income and middle-income countries”. They highlighted the importance of targeting of newborn and child health services to the poor strata of the population, enabling the poor to gain from global efforts to attain the Millennium Development Goals (and now Sustainable Development Goals) [35].

Despite the fact that the inequities and inequalities are declining, the ongoing trend of service coverage for the disadvantaged groups are not at a sufficient pace to accelerate progress towards achieving the goals of universal health coverage (UHC) by 2030, as evidenced by Amouzou et al., and this calls for an urgent need for more robust and effective strategies for equitable access and coverage across all population segments, if the goals for UHC are to be achieved [36].

The National Health Vision for Pakistan (2016–25) mentions the challenges in access in urban areas, and for the poor, and also highlights the issue of inequities in coverage of health services as well as access to healthcare. This vision document encourages equity based and pro-poor approach for all interventions in the health sector, with special emphasis on inequities regarding maternal, neo-natal and child health services. Special initiatives are recommended to be implemented through an equity focused approach, i.e. by specifically targeting the rural areas, urban slums, and the lower segments of the population. Initiatives like provision of round the clock obstetric and neo-natal care at primary level, such as done under the Integrated Reproductive, Maternal, Newborn & Child Health and Nutrition Program (IRMNCHNP) in the Punjab province [22] may be considered for replication in other provinces as well as the improvements observed in maternal and neo-natal health in Punjab are attributed to the interventions done under this program.

The coverage of health services, especially from the perspective of neo-natal health, has improved in the country as evidenced through the PDHS (Table 1). The Government now needs to focus more towards improving the quality of care at primary care level, so that the mortality due to common and easily preventable causes i.e. complication of pre-term birth (mostly hypothermia), sepsis and asphyxia can be minimized [14].

## Conclusion

Presentation of outcomes data, such as neonatal mortality in various wealth quintiles is an effective way to highlight the inequities amongst income groups as it highlights the vulnerable and at-risk groups. It serves as an advocacy tool to not only improve coverage of health services, but also to address the issues of inequity in quality of care in rural versus urban areas. In other countries, rural-urban distribution, or ethnic groups may also reflect similar differences and help in identifying high-risk groups.

## Data Availability

Raw data of Pakistan Demographic & Health Survey is available publicly on the Demographic & Health Surveys website. The same was used for the purpose of this study. The analysis sheets can be shared if required.

## Abbreviations

FATA: Federally Administered Tribal Area
GB: Gilgit Baltistan
ICT: Islamabad Capital Territory
IRMNCHNP: Integrated Reproductive, Maternal, Newborn and Child Health & Nutrition Program
KP: Khyber Pakhtunkhwa
NIPS: National Institute of Population Studies, Pakistan
NMR: Neonatal Mortality Rate
PDHS: Pakistan Demographic & Health Survey
SPSS: Statistical Package for Social Sciences
SDGs: Sustainable Development Goals
UNIGME: United Nations Inter-agency Group for Child Mortality Estimation
USAID: United States Agency for International Development
UHC: Universal Health Coverage
WHO: World Health Organization

## Footnotes

1 *It is pertinent to note that the negative sign just reflects a more favorable situation for the richer quintiles, so it should not be treated as a regular negative number* i.e. *-2 will be a bigger value than − 1 in the case of concentration index.*
